# Solar UV-treatment of water samples for stripping-voltammetric determination of trace heavy metals in Awash river, Ethiopia

**DOI:** 10.1016/j.heliyon.2016.e00091

**Published:** 2016-03-17

**Authors:** Gelaneh Woldemichael, Taffa Tulu, Gerd-Uwe Flechsig

**Affiliations:** a Adama Science and Technology University, Adama, Ethiopia; b Addis Ababa University, Addis Ababa, Ethiopia; c University at Albany, SUNY, Department of Chemistry, 1400 Washington Avenue, Albany, NY 12222, United States; d University of Rostock, Department of Chemistry, A.-Einstein-Str. 3a, D-18059 Rostock, Germany

**Keywords:** Voltammetry, Sample handling, Polarography, Environmental analysis

## Abstract

We report about testing a new mobile and sustainable water sample digestion method in a preliminary field trial in Ethiopia. In order to determine heavy metals at the ultra-trace level by stripping voltammetric techniques in water samples from Awash River, we applied our new method of solar UV-assisted sample pretreatment to destroy the relevant interfering dissolved organic matter. The field tests revealed that 24 h of solar UV irradiation were sufficient to achieve the same sample pretreatment results as with classic digestion method based on intense and hard UV. Analytical results of this study suggest that both a hydroelectric power station and agrichemical applications at Koka Lake have increased the levels of the investigated metals zinc, cadmium, lead, copper, cobalt, nickel, and uranium.

## 1 Introduction

### 1.1 Investigations of environmental pollution in Africa

Heavy metals usually exist only in trace amounts in the environment; however, they may accumulate unnoticed up to worrying levels. The contamination effects of such heavy metals on human health have been well studied in the past. One major source for heavy metal contaminations are mining activities [1].

Such industries have been flourishing in Africa since the colonial ages. Increasing efforts in environmental studies have revealed major problems [2]. The Niger River has been investigated recently, and extreme pollution by heavy metals such as zinc, copper, chromium, nickel and cadmium was confirmed [3, 4]. Also in India, heavy metal concentration in rivers are sometimes extreme with levels up to the mg/L range for cobalt, copper, nickel, zinc, and lead [5].

In spite of relatively low levels of industrial development in many African countries such as Ethiopia, there have been for decades increasing immission loads of toxic heavy metals in the various environment compartments including soil, air and water. Over the last decade, increased activities in both industry and urbanization have led to rising heavy metals inputs all over Ethiopia. In particular, the levels of arsenic, chromium, mercury, cadmium, zinc, and lead are of great concern. Awash River is one of the economically most important rivers in Ethiopia. Awash River originates from the Central Highland of Ethiopia, west of Addis Ababa near Ginchi Town. It flows in southwest direction passing the southern side of Addis Ababa City where it joins Akaki River. The Akaki River originates from Addis Ababa with many other creeks, which are rich in industrial and municipal wastes. Mojo River, another carrier of immense industrial wastes particularly from tannery, joins Awash River near Koka Dam. Awash River continues its course eastward in the Great East African Rift Valley thousands of kilometers across Afar depressions and ends in the Afar desert.

Awash is probably the most economically utilized river in Ethiopia. It provides drinking water to more than five million people living in urban areas like Adama, Wonji, and the surrounding rural populations. Some of the pastoralist populations like Afar and Kereyu are totally dependent on the Awash River for all of their activities. The Awash River irrigates many large-scale farmlands and plantations like Wonji, Nura Era, Merti Jeju, Metehara and a large number of small-scale farms and plantations for local population supporting millions of lives. Awash is the first river on which modern hydroelectric power was build (Koka 1, 2 and 3). Fresh water fishing from the river basin and Koka artificial lake is one of the largest fish suppliers to Addis Ababa, Adama, Bishoftu, Mojo and surrounding towns.

### 1.2 Environmental heavy metal analysis in developing and emerging countries

Trace metal analysis requires techniques that can detect ppb and ppt levels, while coping with sample matrix components like salts and dissolved organic matter. State of the art are therefore most sensitive methods like inductively coupled plasma optical spectrometry (ICP-OES), inductively coupled plasma mass spectrometry (ICP-MS) and graphite furnace atomic absorption spectrometry (GF-AAS) coupled with extraction and enrichment steps. These instrumental techniques are mostly not available at universities and in government laboratories in developing and emerging countries. If atomic absorption spectrometers are available, these are mostly equipped with flame atomization (F-AAS) [3], which requires enrichment of lower ppb traces by water evaporation [5, 6]. This step is usually coupled with open or pressurized nitric acidic digestion procedures [4, 7, 8], which makes the method prone to contamination and increased blank values. This way, trace concentrations in the two-digit ppb range can be determined. However, procedures like these are prone to errors, tedious, time- and energy consuming. If western laboratories are involved, samples are often transported over long distances [9]. The methods and procedures available so far do not allow nationwide environmental monitoring of heavy metal pollution in developing countries.

### 1.3 Voltammetric water analysis assisted by UV digestion

Despite low cost and great performance in trace level metal determination, little has been published about application of stripping voltammetry in developing and emerging countries. However, even this affordable technique requires sample pretreatment by UV oxidation [10, 11, 12]. This technique allows for complete degradation of dissolved organic matter (DOM) that otherwise would disturb voltammetric measurements by complexing metal ions and interfering surfactant properties. At the same time, UV digestion brings only minimal contamination (blank values) by using very small amounts of hydrogen peroxide and nitric acid. A review revealed that usually powerful UV lamps with up to 1000 watts have been used [10]; however, the method was successfully tested even with a lab-made UV irradiation device comprising three 20 watts low-pressure Hg lamps for wine sample pretreatment to allow F-AAS determination of iron and manganese [13].

### 1.4 Solar UV water treatment

The term SODIS (solar water disinfection) designates a procedure that allows disinfection of drinking water by simplest and widely available means [14, 15, 16]. UV-A transparent plastic bottles (mostly PET) are filled with filtrated water from lakes and rivers and left in the sunlight for at least 6 h [17]. During this time, UV-A will cause dissolved oxygen to form active species such as superoxide, ozone and hydroxyl radicals that will inactivate more than 99% of infectious microbes. Addition of H_2_O_2_ and lowering the pH accelerate the process [18]. Literature on this method has been reviewed recently [19].

During the past couple of years we have introduced a method of water-pretreatment that bases on solar UV photooxidation of complexing dissolved organic matter. This way, the most sensitive techniques of stripping voltammetry have been successfully applied to determine Zn, Cd, Pb, Cu by anodic stripping-voltammetry (ASV) [20], as well as uranium [21], Co, and Ni [22] by adsorptive stripping-voltammetry (AdSV) in river water samples of the Warnow River (Germany). At that location, a maximum solar UV-A intensity of 3.5 mW/cm^2^ could be observed. Still, the effect upon complexing dissolved organic matter was remarkable, in particular for adsorptive stripping-voltammetric determination of Ni, Co, and U.

Here we present a pilot field study that aimed at testing the feasibility of the new sample pretreatment method for heavy metal monitoring in the waters of an Ethiopian River. The two objectives were:

a) Optimization of the time required to complete the digestion process of interfering dissolved organic matter (DOM) in the river water samples at locations with relatively high solar UV-A intensity close to the equator followed by systematic evaluation of the results by comparing with pre-treatment by classic UV digestion based on intense hard artificial UV-C.
b) Determination of 7 heavy metals (cadmium, cobalt, copper, lead, nickel, uranium and zinc) in different locations on Awash River, Ethiopia by applying the newly developed sample pre-treatment method.

## 2 Experimental

### 2.1 Sample collection and preparation

**Fig. 1:**
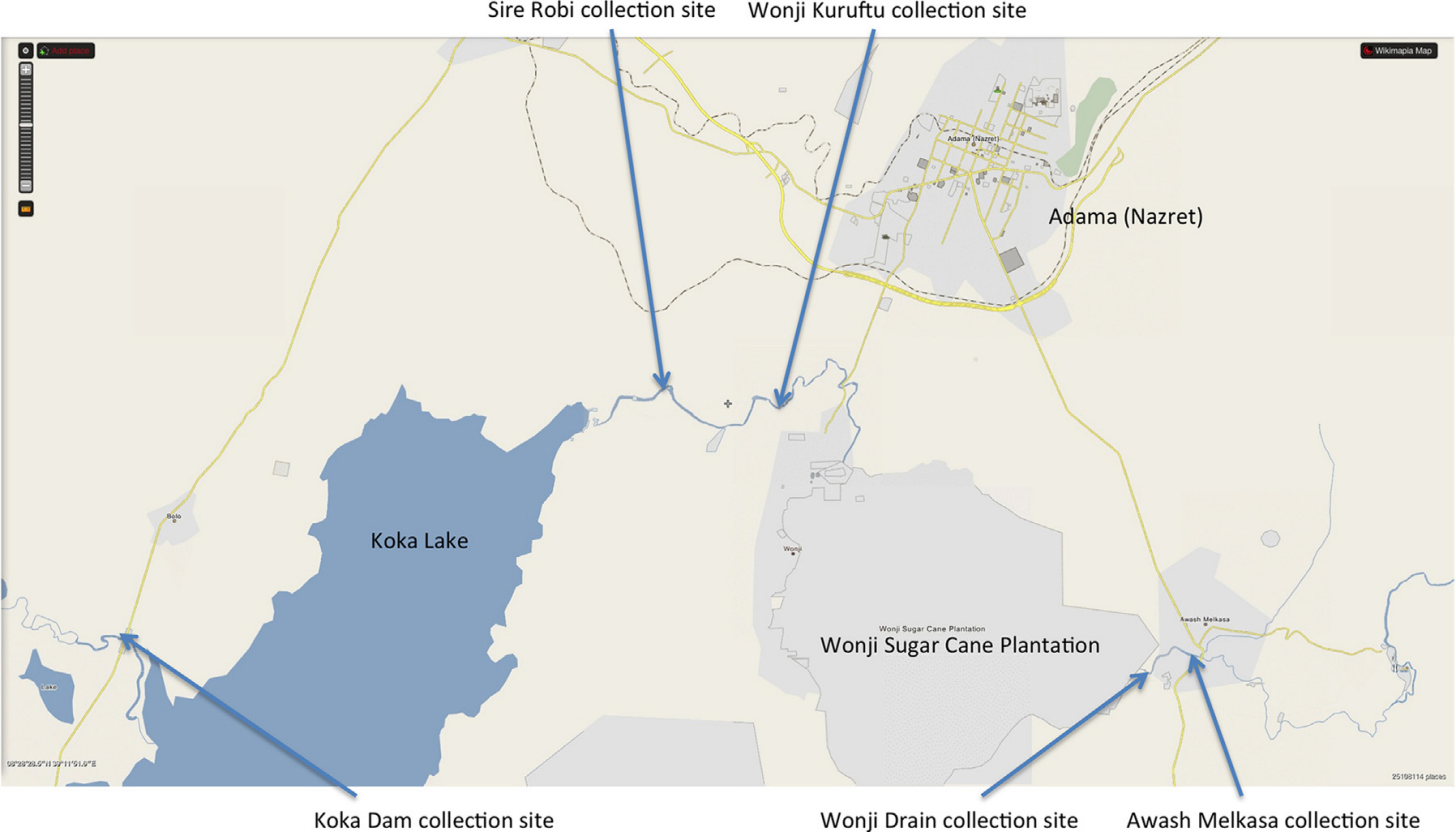
Map of the 5 sampling sites at the Awash River, Ethiopia, reproduced from Wikimapia with permission by license CC-BY-SA.

Water samples from Awash River were collected on March 17, 2012 in Ethiopia, Oromia Regional State, East Shewa Zone at locations given in Table 1 and on the map (Fig. 1). No specific permissions were required for our activities at these public locations, as they are freely accessible. This field study did not involve endangered or protected species. As a sub-equatorial region at an elevated altitude, significantly higher solar UV-A intensity (averaged 5.4 mW/cm^2^ at the time of sampling) than in Rostock (Germany) could be used and tested in this study. The altitude at the sampling sites ranges from 1542 to 1595 meters above sea level. After filtration and acidification to pH 2 with 1 mL HNO_3_ (65%, TraceSelect grade) per liter sample and addition of 100 μM H_2_O_2_, the filtered samples were solar UV-irradiated in thin and clean UV-A-transparent 20 × 15 cm^2^ polyethylene bags for 12 or 24 h as described below. The usual irradiation sessions were scheduled between 10 am and 4 pm due to the maximum sun light intensity. An aluminum reflector was used to increase the irradiation efficiency. All samples were then packed and shipped to the University of Rostock (Germany) for voltammetric determination of trace metals applying analytical equipment and procedures as described below [20, 21, 22]. Before and after this 2-day period of transport, the samples were stored at 5 °C. For the reference measurements with the 24 h SoUV samples, an additional classical UV digestion in quartz glass tubes was performed at Rostock University by means of a 30 watts, 254 nm low-pressure mercury lamp.

### 2.2 Materials

Chemicals were delivered by Merck and Fluka (Germany). Nitric acid (65%), acetic acid, hydrochloric acid (30%), potassium chloride, ammonium hydroxide (25%), and sodium hydroxide were all of TraceSelect analytical grade. Metal stock solutions were purchased as 1000 mg/L AAS standard solutions. All other chemicals were of analytical or reagent grade. Ultrapure water was prepared by means of an SG Water system (2005, SG Water GmbH, Germany, now Evoqua LLC; 18 MΩ*cm, TOC < 2 ppb). Amidosulfonic acid [23] and hydroxylammonium sulfate [24] at a level of 100 mg/L were added, if needed to remove any remaining nitrite after the irradiation step, as this interfering ion could be formed during UV irradiation in the presence of nitrate [25]. The following reagent and buffer stock solutions were prepared: Acetate-KCl buffer (1 M KCl containing 0.5 M equimolar sodium acetate/acetic acid), ammonia buffer (1 M NH_4_Cl, 2 M NH_3_), 0.1 M dimethylglyoxime (DMG), and 5 mM chloranilic acid.

### 2.3 Equipment

Solar UV-A intensity at sampling sites was measured by means of a UV light meter LT Lutron YK-35UV, equipped with a filter covering the range from 290 to 390 nm. Voltammetry was performed using a Metrohm 663 VA Stand equipped with Hg multi-mode-electrode, glassy carbon rod counter electrode and Ag/AgCl (3 M KCl) reference electrode. The 663 VA Stand was controlled by a μAutolab I (Ecochemie B.V., Utrecht, The Netherlands) via an IME interface (Metrohm AG, Herisau, Switzerland). The Autolab was controlled by a PC with GPES 4.9 software. To use the automatic hanging mercury drop electrode mode in this configuration, the rotary switch at the 663 VA Stand was turned to position “SMDE” [sic], as advised by Metrohm.

### 2.4 Voltammetric measurements

Measurements were conducted by inverse (stripping) differential-pulse voltammetry (DPV) at a hanging mercury drop electrode (HMDE) according to the German Industry Standard DIN 38406 parts 16 and 17. The voltammetric parameters are given in Table 2. All samples were purged with nitrogen for 10 min in order to remove oxygen. A 0.05 M acetate buffer with 0.1 M KCl at pH 4.6 was used for the determination of Zn, Cd, Pb, and Cu according to DIN 38406–16. All four metals were accumulated cathodically as amalgams in the HMDE, and then stripped-off in one anodic DPV scan. For the AdSV determination of nickel and cobalt at pH 9.5 according to DIN 38406–16, we added 1 mL ammonia buffer and 2 mL DMG stock solution to 20 mL of the sample. Both metal ions were accumulated as DMG-complexes at the surface of the HMDE, and then stripped-off in one cathodic DPV scan. Determination of uranium by AdSV with 120 μM chloranilic acid as ligand was conducted at pH 2, adjusted by adding nitric acid [26]. The uranium(VI) analyte formed a complex with chloranilic acid that was accumulated by adsorption at the surface of the HMDE, followed by a cathodic DPV stripping scan (DIN 38406–17).

## 3 Results and discussion

### 3.1 Optimization of time of solar UV irradiation and evaluation of solar UV digestion efficacy

One of the main objectives of this study was to investigate the dose of solar UV (SoUV) irradiation necessary to obtain the same analytical results as with the reference method based on hard artificial UV light. Accordingly, original river water samples from Sire Robi collection site have been divided into three aliquots each in order to test the following three different irradiation procedures: a) 12 h SoUV, b) 24 h SoUV, and c) 24 h SoUV + 6 h artificial UV irradiation.

The irradiation procedures of the sample aliquots were carried out based on previously developed methods [20, 21, 22]. The heavy metal concentrations obtained after 12 h SoUV, 24 h SoUV and 24 h SoUV + 6 h artificial UV-irradiated samples are presented in Table 3. These values suggest that 12 h of solar UV irradiation at the above conditions already could degrade large portions of the matrices. However, the decomposition was incomplete and this was confirmed by the results obtained after 24 h SoUV irradiation. As a result of this doubled SoUV dose, the amount of free metal ions available for voltammetric determination increased by 17% for Zn(II), 66.7% for Cd(II), 37.9% for Pb(II) and 37.7% for Cu(II). The regarding results for AdSV of Ni(II), Co(II) and U(VI) revealed an increase by 26.4%, 9.8% and 18.2%, respectively.

In order to evaluate the efficacy of a total 24 h solar UV irradiation, we performed reference measurements with the third aliquot of the water sample from Sire Robi collection site after an additional step of 6 h artificial UV irradiation with a 30 W low-pressure Hg lamp of 254 nm (i.e. equivalent to classic 60 min UV digestion with 180 W lamp). Such additional treatment of the samples revealed no further significant increase of the detected concentrations of the metal ions under consideration (Table 3). This confirms that 24 h of solar-UV irradiation at average intensity of 5.4 mW/cm^2^ or more will lead to sufficient depletion of interfering dissolved organic matter (DOM) in river water. The results of this experiment showed that further hard UV irradiation has no significant effect on the amount of free metal ions available for voltammetric determination of all 7 tested metals in river water or similar other natural water samples.

### 3.2 Analytical performance of the used equipment and method

Prior to the determination of the 7 trace metals in all the river water samples, we examined the method detection limit (MDL) for each metal. This was a necessary step in order to evaluate the range with signal to noise ratio (S/N) greater than 5, i.e. to make sure that the observed concentrations for each trace metal was significantly above the noise level, as the latter would be affected by the particular matrix, protocol, and analyte. The investigated MDL values are presented in Table 4 in comparison with instrumental detection limits (IDL) advised by the manufacturer of the instrument (Metrohm-Autolab). These results demonstrate that the MDL values of our voltammetric protocols for all the investigated metal ions were well below the concentrations found in the river water samples, with one exception: The cobalt(II) level in two samples was found to be below our MDL (Table 6 ). This allowed us to apply our protocols for the monitoring of 7 trace heavy metals in Awash River even at the ng/L level.

### 3.3 Toxicological review of Awash river in respect to cadmium, cobalt, copper, lead, nickel, uranium and zinc

The analytical results of the samples collected from different sites of Awash River were employed to evaluate the degree of contamination of the river with respect to the target heavy metals based on international (WHO) water quality standards. We also evaluated the status of health hazards (risks) associated with heavy metal pollution of Awash River. In general the determination of these heavy metals helped us to analyze toxicological impacts related to this major heavy metals contamination.

The analytical results of the 7 heavy metals under consideration of the two closest sampling sites upstream and downstream of the Koka reservoir and the Power Station are summarized in Table 5. All analytical results were corrected for the observed background concentrations (blank values) caused by all possible contaminations (following the sampling) such as reagents, plastic bottles (sample containers), polyethylene bags for irradiation, the filter paper, the electrochemical cell, and the electrolyte buffer. Critical evaluation of the variation of the concentrations of the samples collected from Koka reservoir and Sire Robi is demonstrated in Table 5. This investigation reveals that the concentrations of Cu(II), Pb(II), Ni(II), U(VI) and Zn(II) increased downstream by 45.4%, 25.9%, 28.4%, 3.0% and 8.9%, respectively. On the contrary, the concentrations of Cd(II) and Co(II) decreased downstream by 3.5% and 4.9% respectively. Koka Dam samples were collected from the upstream of the Dam just 200 meters before the river joins Koka artificial lake. The time of sampling corresponded to the dry season at that location implying relatively low flow rate, and water volume, and hence, a high DOM content. The other collection site (Sire Robi) is located downstream beyond the outlet of the dam (about 3 km from Koka hydroelectric power station) just at the Adama drinking water treatment plant. The rationale behind the decrease/or increase in the concentrations of metal ions downstream can be explained by considering various factors. For instance, the physical and chemical processes taking place in the water; the velocity (flow rate) of the water; turbulences; water volume; and landscape. Aquatic fauna and flora are some of the factors that affect migration of the metal contaminants and the matrices or the fate and transport of the complexing DOM, and hence, the metal ions. There can be significant number of flora in the reservoir feeding on Cd(II) and Co(II). This needs further investigation. In this particular case, the velocity and turbulence of the water decreases quickly as soon as the river enters the lake.

The observed small variations for Cd(II), Co(II), and U(VI) levels were below 5%, and can probably be attributed to the usual variations of manual sample collection. On the contrary, the concentrations of Cu(II), Pb(II), Ni(II) and Zn(II) significantly increased at the downstream collection site. These metals are all found in alloys like brass and bronze as used in components (bearings) of power plant machineries. One of the most probable causes, therefore, could be the interaction of the river water with such metal components, lubricants and other materials such as paints at the hydroelectric power station.

Table 6 presents a comprehensive view of all investigated analytes and sampling sites. The agrochemicals used by the local farmers could be another source of contamination. Artificial phosphate fertilizers for instance, are known to contain relatively high uranium levels [27]. The nearby Tannery (Ethiopian Tannery) also discharges its waste into the lake. This deadliest waste can contribute to the unexpected sharp increase in the concentration of some metals. In addition to these, there are extensive horticultural fields, which use agrochemicals intensively. The leaching of these chemicals into the lake could lead to the increase of the concentration of the metals by polluting the river water. However, the reason why the concentration of the metal pollutants increased downstream requires further investigation to identify the point and non-point sources and to recommend possible solutions.

Further investigation also revealed that some metal ions increased in concentration much more than expected while others decreased downstream. For instance Zn(II) increased from 126.62 to 440.85 μg/L as we move from Koka Dam (upstream Koka Lake) to Awash Melkasa. On the contrary, the Cu(II) concentration decreased from 25.45 to 6.25 μg/L in the same direction. In general, the determination of the concentration of these heavy metals helped us to analyze the degree of pollution and hence their toxicological impact on humans and animals based on the WHO safe drinking water guideline quality standards (Table 7). Deep investigation of the concentration data of the metal ions in the river would lead to the conclusion that it is moderately risky for drinking with respect to lead, while it is safe to drink this water with respect to cadmium, copper, nickel, cobalt, uranium and zinc. In conclusion, local people living around Awash River and the Adama City dwellers who drink water from Awash River and Koka Lake are at moderate risk of lead intake and the regarding health hazards. We suggest further studies to identify the root causes of the contamination and to recommend remedies.

## 4 Conclusions

This study has demonstrated that solar UV irradiation assisted by low pH and addition of hydrogen peroxide can serve as a means for water sample digestion. A solar irradiation time of 24 h in equatorial regions is sufficient to allow accurate voltammetric metal determination. This was evaluated by reference measurements including an additional classic UV digestion step with hard and intense artificial UV light (254 nm, 30 W, 6 h). Affordable UV-transparent materials such as thin polyethylene bags are suitable sample containers during irradiation. The presented protocol can be recommended for ASV determination of trace elements such as cadmium, copper, lead and zinc, as well as AdSV of nickel, cobalt, and uranium in river water and similar natural water samples at remote places, where high-power electric UV lamps or commercial UV digestion units cannot be used or made available. The effect of UV irradiation treatment for nickel, cobalt, and uranium was even larger compared to anodic stripping voltammetry of the other metals in the same river water samples, as reported earlier. The approach reported here should be very useful for mobile electrochemical heavy metal analysis in regions where frequent sunny weather conditions are available as proved by the investigation of Awash River water samples from selected locations in Ethiopia. The method is applicable in real sample analysis of heavy metals contamination. This preliminary field study suggests that Awash River water is moderately risky for drinking with respect to lead and cadmium, while it is in the range of safe drinking water with respect to copper, nickel, cobalt, uranium, and zinc. Future developments of our approach will aim at replacement of the HMDE with bismuth electrodes, as well as shortening of the solar irradiation time and development of solar-powered automatic monitoring stations.

## Declarations

### Author contribution statement

Gelaneh Woldemichael: Conceived and designed the experiments; Performed the experiments; Analyzed and interpreted the data; Wrote the paper.

Taffa Tulu: Analyzed and interpreted the data; Wrote the paper.

Gerd-Uwe Flechsig: Conceived and designed the experiments; Analyzed and interpreted the data; Contributed reagents, materials, analysis tools or data; Wrote the paper.

### Funding statement

This work was supported by Ministry of Education of Ethiopia, Adama Science and Technology University and German Academic Exchange Service, DAAD (Engineering Education Capacity Building Program, EECBP), as well as German Research Foundation (DFG Heisenberg Fellowship, FL 384/7-2).

### Competing interest statement

The authors declare no conflict of interest.

### Additional information

No additional information is available for this paper.

## Figures and Tables

**Table 1:** Sampling sites, Awash River, Ethiopia.

Sampling site	GPS coordinates	Altitude (m)
Koka Dam	8°24′24.02″N, 39°01′11.66″E	1595
Sire Robi	8°28′45.82″N, 39°10′52.41″E	1553
Wonji Kuruftu	8°28′24.37″N, 39°12′47.98″E	1553
Wonji Drain	8°23′39.38″N, 39°19′16.11″E	1545
Awash Melkasa	8°24′04.91″N, 39°20′03.28″E	1542

**Table 2:** Differential-pulse stripping-voltammetric parameters for ASV of Zn(II), Cd(II), Pb(II), Cu(II), and AdSV of Ni(II), Co(II) and U(VI) in Awash River water samples.

Voltammetric parameters	Analytes		
Zn(II), Cd(II), Pb(II), Cu(II)	Ni(II), Co(II)	U(VI)
Method	ASV	AdSV	AdSV
Deposition potential (V)	-1.15	-0.7	0.1
Deposition time (s)	120	120	30
Equilibration time (s)	10	5	5
Modulation amplitude(V)	0.0202	0.0202	0.05
Modulation time (s)	0.05	0.05	0.05
Interval time (s)	0.5	0.10	0.10
Start potential (V)	-1.15	-0.8	0.04
End potential (V)	0.05	-1.2	-0.21
Voltage step(V)	0.049	0.049	0.006
Scan rate (V/s)	0.01	0.013	0.012

**Table 3:** Effect of UV irradiation procedure upon the determined heavy metal concentrations in Awash River water samples collected from Sire Robi.

UV irradiation	Concentration (μg/L)						
Zn(II)	Cd(II)	Pb(II)	Cu(II)	Ni(II)	Co(II)	U(IV)
12 h SoUV	115.89	0.12	3.06	26.61	4.66	0.51	0.18
24 h SoUV	135.53	0.20	4.22	36.65	5.89	0.56	0.22
24 h SoUV + 6 h UV	137.85	0.20	4.24	37.01	5.97	0.55	0.22
% increase due to additional SoUV irradiation	16.9	66.7	37.9	37.7	26.4	9.8	22.2
% increase due to additional UV irradiation	1.02	0	0.47	0.98	1.36	0	0

**Table 4:** Instrumental detection limits (IDL) of the manufacturer and method detection limits (MDL) determined according to the procedure given in experimental section.

Analyte	IDL (μg/L)	MDL(μg/L)	Spiked concentration (μg/L)
Cadmium	0.050	0.073	0.100
Cobalt	0.050	0.089	0.100
Copper	0.050	0.260	0.100
Lead	0.050	0.240	0.500
Nickel	0.050	0.058	0.100
Uranium	0.025	0.120	0.100
Zinc	0.050	0.274	1.000

**Table 5:** Comparison of percentage variation of the concentration of heavy metals of two sampling sites upstream and downstream of Koka power station.

Sampling Site	Metal ion concentrations (μg/L)						
Zn(II)	Cd(II)	Pb(II)	Cu(II)	Ni(II)	Co(II)	U(VI)
Koka Dam (upstream)	126.62	0.21	3.37	25.45	4.67	0.57	0.19
Sire Robi (downstream)	137.85	0.20	4.24	37.01	5.97	0.55	0.22
Concentration Variation (downstream) %	8.86	-4.84	25.85	45.42	28.36	-3.52	3.00

**Table 6:** Variation of the concentration of heavy metals in Awash River.

Element	Background concentrations (μg/L)	Background-corrected sample concentrations (μg/L)				
Awash Melkasa	Wonji Drain	Wonji Kuruftu	Sire Robi	Koka Dam
Zn	28.09 ± 0.86	**441** ± 11	26.12 ± 2.62	76.70 ± 7.11	137.9 ± 1.60	126.62 ± 1.13
Cd	ND^a^	**2.87** ± 0.05	0.06 ± 0.02	0.11 ± 0.03	0.20 ± 0.01	0.21 ± 0.03
Pb	1.59 ± 0.05	**10.93** ± 0.50	0.52 ± 0.05	1.14 ± 0.21	4.24 ± 0.09	3.37 ± 0.17
Cu	6.25 ± 0.72	4.33 ± 0.06	11.70 ± 0.80	25.71 ± 2.27	**37.0** ± 3.7	25.45 ± 0.56
Ni	2.21 ± 0.01	0.75 ± 0.09	3.35 ± 0.51	**20.44** ± 0.29	5.97 ± 0.94	4.67 ± 0.45
Co	ND^a^	ND^a^	0.21 ± 0.06	ND^a^	0.55 ± 0.03	**0.57** ± 0.04
U	0.84 ± 0.01	**1.41** ± 0.17	0.56 ± 0.47	1.35 ± 0.21	0.22 ± 0.13	0.19 ± 0.25

Maximum values highlighted.

a Not detected, i.e. below the method detection limit.

**Table 7:** Heavy metals’ pollution status in Awash River according to World health organization (WHO) Guidelines for Drinking-Water Quality, Geneva.

Element	Level in drinking water (μg/L)	Maximum observed concentration (μg/L)	WHO guideline value (μg/L)	Toxicological evaluation
Cadmium	1.0	2.87	3.0	endangered
Cobalt	Not specified	0.57	Not specified	safe
Copper	5–30,000	37.0	2000.0	safe
Nickel	<20.0	20.44	70.0	safe
Lead	5.0	10.93	10.0	endangered
Uranium	<1.0	1.41	15.0	safe^a^
Zinc	10.0–50.0	441	3000.0	safe

a Guideline value for tap water in Germany is now 10 μg/L, whereas maximum 2 μg/L has been recommended for infants (both tap and mineral water) [28].
